# Deeping Our Relationships for Healing: Our Land, Our People, Our Freedom

**DOI:** 10.1089/heq.2022.29024.soc

**Published:** 2023-01-20

**Authors:** Lisa Sockabasin

**Affiliations:** Wabanaki Public Health and Wellness, Bangor, Maine, USA.

The relationship with Indigenous people and the United States is devastating and complex. There is shame carried within this relationship. The shame holds the power to force society, systems, and individuals to create new narratives. Narratives that both ignore the brutal past and devalue the gifts of the people forcibly violated. Thus, creating a society that hides its past and embeds privilege and value for some, while dehumanizing and making others invisible. For Indigenous people, this invisibility has deep impact, one of which is generational health disparities. Indigenous people are highly visible in statistics of despair, such as violence against women, murder, infant mortality, neonatal abstinence syndrome, and more.

The health statistics for Indigenous people are often not coupled with the stories of why such despair exists. The stories of shame have been erased from our textbooks but not our bodies, a new narrative created, resulting in irreparable harm to Indigenous people. This harm, however, is not isolated to Indigenous people, the harm is shared with all people. The absence of indigenous ways of being, knowing, and connecting has immense impact on our nation's ability to live in relationship with this land and with each other.

There is great divisiveness in our world and country today. This divisiveness can be easily seen with a quick social media scroll or a click on the television. This divide does not serve us well as people or communities. Indigenous values bring people together, across difference. Our land is also witness to the loss of indigenous values. The value of holding the land with care and reciprocity has been missing in mainstream society for far too long. The lack of indigenous worldviews in mainstream society is a society that lacks connection and respect for all, a society that values individualism rather than collectivism. Indigenous values express the importance of respecting the sovereignty of the land and her people: viewing the land as a sacred partner, rather than an object of possession and control.

As a partner, the land and her people serve each other, providing nourishment collectively. The loss of indigenous voice and leadership across America has created a great imbalance. We have become a nation of people believing in invisibility and the retelling of history, a place where divisiveness is dominant and seeing truth is too painful. Although Indigenous people have paid the highest price, all others who share this land must understand the devastation will be and is being shared with each of us.

## A Framework: A Plan, a Path Forward

Race, power, and privilege have a variety of meanings to each of us and elicit a diversity of emotional responses. In America, Indigenous people have lived experiences with this country like no other people sharing this land. Our collective lived experiences in America are vastly different, and these differences are important. Some experiences are a great reminder of the harsh realities of our history, and some a great reminder of how history comfortably instills hope and privilege in others. Americans and those who now live here share a society that places higher value on some lives over others.

Today, we have a longstanding epidemic of missing and murdered Indigenous women and girls that is rarely reported on mainstream media or discussed in governmental or academic settings, while Indigenous women and girls go missing or murdered at rates higher than any other demographic. “Native women are 2.5 times more likely to be sexually assaulted than any other demographic of women in the country,” I found a team of Native researchers headed by Crystal Echohawk. This team organized under the project “Reclaiming Native Truth” released a report that revealed for the first time how the American public views Native Americans.

The study found that the largest barrier to public sympathy for Native rights was “the invisibility and erasure of Native Americans in all aspects of modern US society.”^1^ Not only is violence a common experience for Indigenous communities across the nation, access to the most basic human needs is also a struggle. There are Indigenous communities struggling today in 2022 for access to clean drinking water. Some tribal nations hold generations of their people exposed to dirty drinking water, with long-term exposure risks unknown.^2^ Many Americans live in places where boil water orders and water distribution centers are issued at first notice of a water quality concern, while there are Indigenous people and communities where water concerns have been ignored for decades.

The challenges of and steps to address racism and racial inequities are complex to understand and to overcome. The Truth, Racial Health, and Transformation (TRHT) framework allows us to examine the entanglement of race/ism, healing, and meaningful relationship building. The TRHT is a comprehensive process to plan for and address the history and contemporary effects of racism. The TRHT framework centers its work on five key pillars: (1) Narrative Change, (2) Racial Healing and Relationship Building, (3) Separation, (4) Law, and (5) Economy.^3^ The framework helps to facilitate healing from a history often hidden, and a present day paralyzed with disconnection.

Narrative Change and Racial Healing and Relationship Building are foundational pillars. These foundational pillars allow for sharing stories of both sorrow and strength, allowing for the truth to become visible and healing to happen. The Separation, Law, and Economy pillars address how America has historically sustained racial hierarchy and provides a path forward to disrupt structural and systemic racism.^4^ Collectively, the pillars of the TRHT framework support a process that ensures learning from the past and acknowledges the impact of the devastating mistakes made.

Indigenous teachings and values of forgiveness, relationship building, and repair are important for all to consider. Rebuilding our relationships and connecting across difference can deepen the healing journey. These values bring us closer to healing. Recognizing the importance of each of the THRT framework pillars will make this healing even more profound.

## Deepening Our Connections, Our Relationships: Indigenous Understandings

Today and for thousands of years, Indigenous people live(d) with reciprocity and gratitude for the land and community. Forced colonization and assimilation disrupted these ways of being and replaced them with unsustainable and harmful methods of living, attempting to erase indigenous systems with proven efficacy. These same systems innovatively customized to better fit our current evolving complex world. The attempted erasure of these systems is destructive for all people and the planet we share. Indigenous worldviews and values have worked, are working, and will work again, for not just Indigenous peoples, but also for all people. Through the creation of sacred spaces, we can strengthen relationships, understanding, and healing.

By centering traditional knowledge and culture, we can relearn and implement those systems not always practiced but known. By centering indigenous culture and respect for the land, we can expand our understanding of the interconnectedness of all things, fostering greater empathy to shrink the vast divide in America. As former academician and author, Roman Krznaric describes in his book, Empathy *Why it Matters, and How to Get it*, empathy has the power to transform individuals and facilitate social and political change.^5^ The author further describes empathy as providing a revolution of human relationships: relationships and reciprocity, both core responsibilities for many Indigenous people.

America and her people need an infusion of empathy and Indigenous people could be the teachers: teachers, whose wisdom we listen to and voices we amplify. Empathy allows for the divisiveness to soften and for the smiles to appear. It allows us to see the humanity of all people. Empathy is rooted within indigenous values, ceremonies, language, and all ways of being. Fundamental to indigenous culture is the care for all living things and the recognition of the interconnectedness and interdependency of each component of life. A belief held sacred is that we need each other, that each of us holds a piece to the advancement of the whole.

This belief creates a sense of deep belonging, that each life matters and is needed. Each person, community, and living being has value, must be honored, and respected. This belonging provides the motivation to be responsible to both you and the larger community and society. Thus, creating a culture of radical acceptance, visibility for all, and gratitude for each role that collectively results in a nourished and abundant nation.

## The Wabanaki Way: The People of the Dawn

The Maliseet, Mic Mac, Passamaquoddy, and Penobscot Tribes, collectively known as Wabanaki, are the people of the Dawnland, or the people who welcome the first light. Many Wabanaki people greet the first light of the day with ceremony, some may burn smudge (such as sage and sweetgrass), some may offer prayer or song, setting healing intentions for the day and for all those across Turtle Island (North America). This relationship with the land and for the people sharing this land is one that spans greater than 10,000 years. This length of time only begins to quantify the depth of connection Wabanaki people hold for this territory, a place that is now called Maine.

These connections Wabanaki people have proven vital, providing how-to live-in reciprocity with the land, how to care for her, and how she cares in return. This relationship, an unspoken unwritten contract, is unbreakable due to the unwavering commitment to reciprocity and a knowing that there are results with each action. Instructions are held within Wabanaki languages, ceremonies, and culture- providing knowledge and protection for not only Indigenous people, but also for all people who call Wabanaki territory home. It is critical to recognize that for centuries, Wabanaki tribes have been threatened by an accelerated loss of language and culture.

**FIG. 1.**
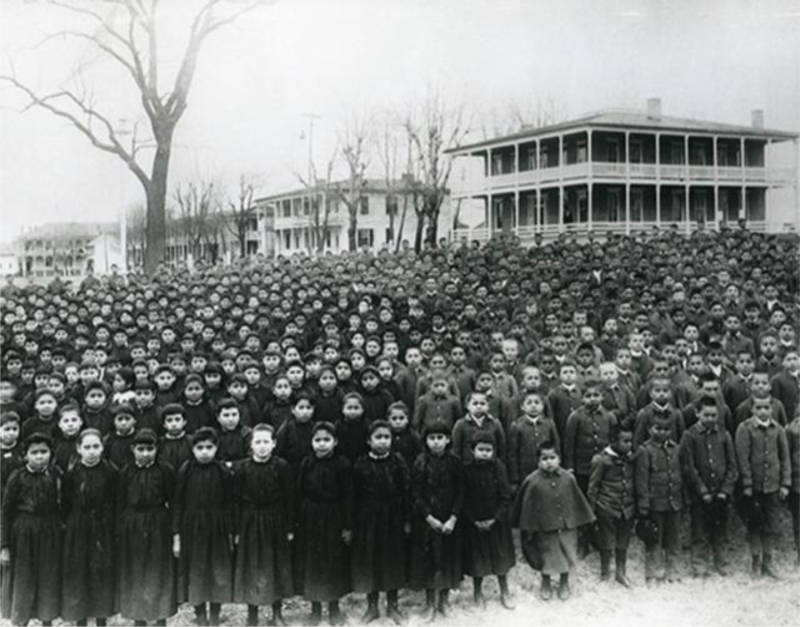
Carlisle Indian Boarding School, March 1892.

The reasons include a variety of factors, such as, early contact and disease, ongoing trauma, poverty, government attempts to acculturate, disproportionate rates of displacement through child welfare removal^6^ and institutional level abuses.^7^ The loss of Wabanaki children by unjust child welfare removal and catholic boarding school efforts, has left wounds deep within the fabric of Wabanaki communities. Wabanaki people are experiencing lowered life expectancy and disparate health status with high incidences of disease that are disproportional to any racial or vulnerable group in Maine^8^ (Fig. 1).

The stories of Wabanaki and other Indigenous ancestors must be told for healing to happen. Truth told and a recognition of the assaults to Indigenous people throughout history and present day, is a story to share and learn from. This story has left Wabanaki and other Indigenous people with the catastrophic consequences of others brutal behavior. Today, complexities are many for Wabanaki people and what is needed, is already deep within; within culture, within ceremony, within language. Historically, Wabanaki Tribes are matriarchal and deeply connected to the land around them. Wabanaki Tribes understand both the fragility of individuals and the strength of the collective.

**FIG. 2.**
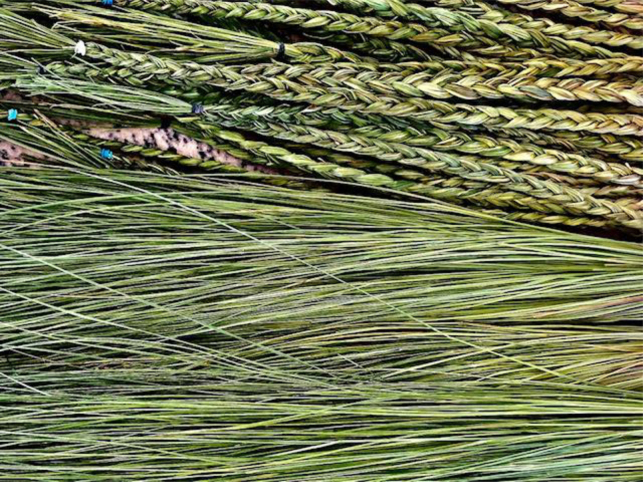
Sweetgrass: Strands and braids, photo by Lisa Sockabasin, Passamaquoddy, August 2021.

This understanding ensures the collective always protects what is sacred—the land, the culture, the languages. The relationship between the three is strong. It is a strength unbreakable, providing all the instructions for abundance, deepening relationships, and enduring difficult complexity: a strength like blades of sweetgrass, one of the Wabanaki sacred medicines, braided together in an everlasting beautiful structure. Wabanaki people's connection to Mother Earth, to culture, and to language can be representative of braided sweetgrass. The tight connection between all the strands woven together to form a braid represents the strength, responsibility, and instructions we all hold (Fig. 2).

## Creating a New Indigenous System for Healing: Wabanaki Public Health and Wellness

The American experience with systems that are meant to serve us is highly variable. Systems of government, health care, education, finance, law, and others were never meant to benefit Indigenous and other people of color. These systems have stories to share, and these stories hold incalculable harm. The systems that were meant to serve so many have paralyzed Indigenous people, making progress painfully slow. Ibram X. Kendi explains in his book, *How To Be* An Antiracist, that all too often we focus on the perceived failings of people rather than the failure of policies that trap them. Kendi's definition of policy includes “written and unwritten laws, rules, procedures, processes, regulations, and guidelines that govern people.”

Kendi further explains that “there is no such thing as a nonracist or race-neutral policy. Every policy in every institution in every community in every nation is producing or sustaining either racial inequity or equity between racial groups.”^9^ Examining and adapting current systems and policies are our collective responsibility. Defining what is working, what does not, and what is needed is a continuous process. Along with this examination, we must also consider creating organizations that build new systems of service and where policies propel healing. As we look into the future, the creation of organizations that heal not only the people they serve but also those providing the service must be top of mind.

Furthermore, implementing healing policies that provide employees a pathway to balance and fulfillment is a wise investment. Healing policies will mitigate the risk of current and future workforce shortage challenges. Generous paid time off, humane wages, retirement opportunities, flexible schedules, career advancement, education and training incentives, and comprehensive health, dental, and eye coverage are all crucial offerings when developing a healing organization.

Wabanaki Public Health and Wellness (WPHW) is a rapidly growing nonprofit organization that provides healing through policies, employment, and service. WPHW serves Indigenous people and the five Wabanaki communities in Maine, Micmac Nation; Houlton Band of Maliseet Indians; Passamaquoddy at Pleasant Point; Passamaquoddy at Indian Township; and Penobscot Nation. The organizational mission is to provide community-driven, culturally centered, public health, social, and healing services to all Wabanaki communities and Indigenous people while honoring Wabanaki cultural knowledge, cultivating innovation, and fostering collaboration.^10^

The organization is building a new system for healing, one that centers values of love, reciprocity, service, and respect for all things living. WPHW is known for hiring passion, not position. It is a place where dreaming daily is not only encouraged but required. Dreaming is woven into daily practice. WPHW works hard to create sacred healing space where dreaming, healing, and service to others is prioritized and practiced. In recent years, the organization has experienced great success and rapid growth. Organizational leaders attribute this growth to the centering of Wabanaki values and the strong belief that all things are possible.

**FIG. 3.**
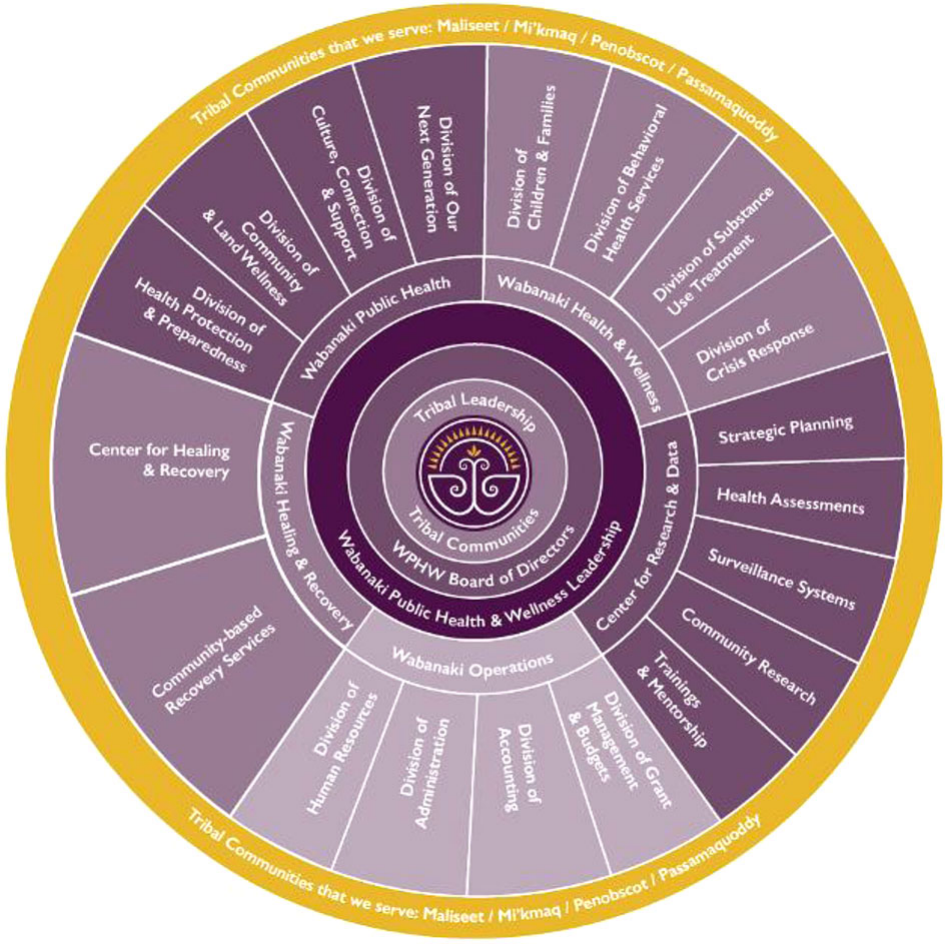
WPHW *Organizational Chart,* April 29, 2022 (internal communication). WPHW, Wabanaki Public Health and Wellness.

WPHW has grown from less than one dozen employees just 4 years ago to close to 150 employees today: a growth rate that is rarely seen and that is described as unbelievable to some. The WPHW team is diverse, with 70% of their workforce indigenous. This large diverse workforce allows for the delivery of a continuum of healing services for a population who has gone far too long without. With just over 13,000 Indigenous people in Maine living statewide,^11^ WPHW works hard to serve in all corners of Wabanaki territory by offering a myriad of healing, recovery, and cultural services (Fig. 3).

## Creating Sacred Spaces: Deepening Relationships and Healing

Dr. Maria Yellow Horse Braveheart describes historical trauma as the “collective emotional and psychological injury both over the lifespan and across generations, resulting from a cataclysmic history of genocide.”^12^ This devastating injury has created an ever pressing need to design sacred spaces for healing, connecting Indigenous people and others to land and culture. The Center for Wabanaki Healing and Recovery, a department within WPHW, is a multiacre and multisite campus offering healing. A healing center focused on reconnection to land, culture, and comprehensive recovery services.

The healing center supports Indigenous people with substance use disorder and their families wherever they are at. WPHW describes its responsibility and commitment to healing this way, “wherever you are on your journey, we have a place for you.”^13^ No matter what, WPHW holds people with love and support, assisting the healing journey to move forward. This recovery system is deeply rooted in connections to natural healing opportunities, such as culture, language, and nature. The Center for Wabanaki Healing and Recovery provides supportive and sacred spaces for healing: spaces that offer connections to Mother Earth and to traditional medicines, foods, ceremony, and recovery programming.

Many of these programs and spaces, once created, work to eliminate health disparities, repair communities, and protect tribal people as they heal. The Center for Healing and Recovery recognizes the deep pain and trauma associated with the history that has remained mostly hidden. The history provides an explanation of the statistics known and the lives lost. It is a history gruesome with details that has impact on indigenous well-being today, even hundreds of years later. Although acknowledging this history is important, equally important is recognizing the resiliency and strength embedded in Indigenous communities.

WPHW defines generational strength as “the power held by our ancestors and passed to us, resulting in a deep knowing on how to connect and how to heal, providing us all we need to know to thrive.”^13^ WPHW works in partnership with Tribes, community members, elders, and others to create the environment where no person is left behind, invisible, or forgotten. WPHW believes in making mistakes, offering forgiveness, learning from difficulty, and that healing is possible for all. There is a strong belief at WPHW that accountability does not have to be scary or dehumanizing, but, rather, offers an opportunity to learn, grow, and make amends.

**FIG. 4.**
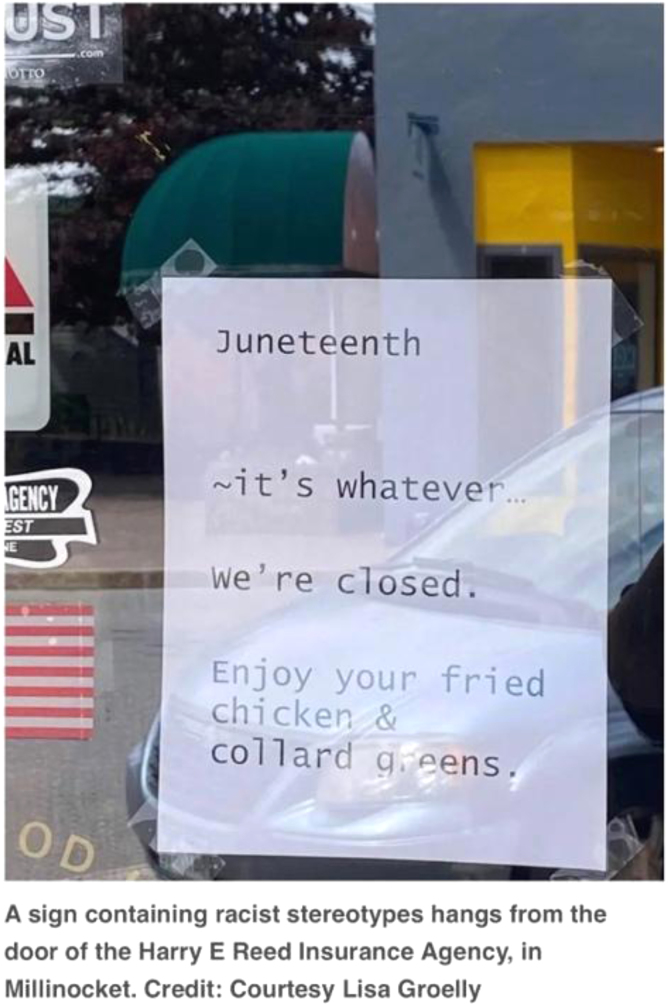
Photo from a business in Millinocket regarding the Juneteenth holiday. *Bangor Daily News*, June 23rd, 2022.

In June 2022 in Millinocket, Maine, an incident of public racism occurred during the week of the Juneteenth holiday. The incident included an offensive sign, placed in a downtown business storefront, highly visible to all who walked by (Fig. 4).^14^ The sign depicted racist stereotypes and disrespectfully made recognition of the Juneteenth holiday. The sign read “*Juneteenth ∼its whatever… We're closed. Enjoy your fried chicken & collard greens*” (Fig. 4).^14^ People within Millinocket, Maine, and nationwide were outraged and expressed anger, disappointment, and frustration.

Many people expressed embarrassment and sorrow that their community was in national headlines for an incident that did not reflect the values of their community. Many other people expressed rage by making phone calls, writing letters, and demanding the permanent closure of the business. This demand was met, and the business is currently in the process of closing their doors for good: one less business in a struggling rural Maine town, with no repair of relationships or opportunity for transformational learning. This type of accountability is dangerous, it offers shame as a motivator, when shame prevents movement and learning.

Creating environments that motivate others to learn and to make amends is the strength of an indigenous worldview. A worldview that does not excuse harmful behaviors but calls for holding sacred space for accountability and deeper understanding. Millinocket, Maine, is the home to the Center for Wabanaki Healing and Recovery. Millinocket, to Wabanaki people, is a place that is sacred, healing, and magical. It is the home to Mount Katahdin, a mountain Wabanaki people have held sacred for thousands of years. This place of healing for Wabanaki people is now loved by many others calling Millinocket home.

**FIG. 5.**
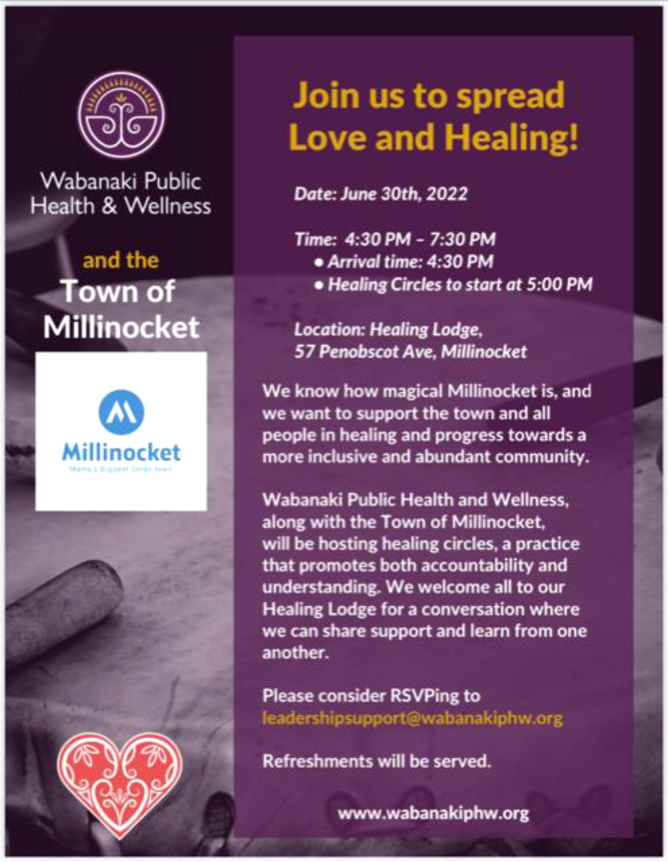
WPHW flyer.

The commitment that Wabanaki people have to this territory extends beyond the land and reaches the people. The Center for Healing and Recovery also believes that where there is divisiveness and pain, Wabanaki people can provide gathering and healing. Where there is darkness, Wabanaki people can provide light, and that light is love—a value that is core to WPHW's work. When WPHW learned of the “sign” incident, the organization quickly sprang into action, providing an opportunity for both healing and accountability. WPHW offered a model for healing and accountability, the healing circle (Fig. 5).^15^

The healing circle is a method of communication that allows for respectful dialogue, learning, and accountability, all while deepening the relationships between others. Healing circles welcome all to share sacred space, ceremony, and prayer—even those who have caused harm. Indigenous values include offering opportunities for reconciliation and repair, rather than humiliation and shame, believing that we do our best learning when we are healing not harming.

The evening of June 30, 2022, less than 1 week after the sign was hung, a Wabanaki healing circle event, in collaboration with the Town of Millinocket, occurred. The event was attended by town officials, tribal elders, business owners, nonprofit leaders, and concerned community members. The event offered ceremony and healing for all. This healing so powerful that it is still being felt in the town today, for those who were negatively impacted by the sign and for those who created it.

## Thinking Ahead: Continuing to Move Toward Healing

The conflicts present in America today seem daunting and unrelenting. While deepening our relationships across differences is crucial, it is also important to recognize that it is often difficult and not pain free. The consideration of indigenous values and worldviews will move us closer together as a nation. These worldviews can offer us opportunity for transformative healing. The indigenous voice has been absent from spaces and decision-making places for far too long. This absence creating risks for all people who share this land.

## Abbreviations Used

TRHT: Truth, Racial Health, and Transformation
WPHW: Wabanaki Public Health and Wellness

## References

[1] Nagle R. Research Reveals Media Role in Stereotypes About Native Americans. Women's Media Center. July 18, 2018; Media, Race/Ethnicity [About 3 Screens]. Available from: https://womensmediacenter.com/news-features/research-reveals-media-role-in-stereotypes-about-native-americans#:~:text=The%20study%20found%20that%20largest%20barrier%20to%20public,K-12%20education%2C%20pop%20culture%2C%20news%20media%2C%20and%20politics [Last accessed: August 2022].

[2] Feinberg R. Passamaquoddy Tribe Hopes New Solutions—And Sovereignty—Can Help Address Longstanding Water Problems. Maine Public. July 22, 2021; The Rural Maine Reporting Project [About 4 Screens]. Available from: https://www.mainepublic.org/health/2021-07-22/passamaquoddy-tribe-hopes-new-solutions-and-sovereignty-can-help-address-longstanding-water-problems?_amp=true [Last accessed: August 2022].

[3] Woods Fund Chicago. Truth, Racial Healing, and Transformation (TRHT). Chicago, IL; Updated December 1, 2018. Available from: https://www.woodsfund.org/news/truth-racial-healing-and-transformation-trht?msclkid=139b3361d07111eca1a29bc76c0e7ce7 [Last accessed: August 2022].

[4] Christopher G. Introduction—Healing through policy: Creating pathways to racial justice. Natl Civ Rev 2021;110(3):6–10.

[5] Krznaric R. Empathy Why It Matters, and How to Get It. Penguin Group: New York; 2014.

[6] Wabanaki REACH. Maine Wabanaki-State Child Welfare Truth and Reconciliation Commission Findings; 2015. Available from: https://d3n8a8pro7vhmx.cloudfront.net/mainewabanakireach/pages/1584/attachments/original/1617238922/FINAL_REPORT_-_FINDINGS.pdf [Last accessed: August 2022].

[7] Wright V. Shattered. DownEast. n.d. Available from: https://downeast.com/features/wabanaki-people-shattered/ [Last accessed August 2022].

[8] Wabanaki Public Health. 2010 Waponahki Tribal Health Needs Assessment. Bangor, ME; 2015.

[9] Kendi I. How to Be an Antiracist. Penguin Random House LLC, New York: 2019.

[10] Wabanaki Public Health and Wellness. Mission and History. Bangor; n.d. Available from: https://wabanakiphw.org/about/mission-and-history/ [Last accessed: August 2022].

[11] United States Census Bureau. My Tribal Area. Available from: https://www.census.gov/tribal/ [Last accessed: August 2022].

[12] Yellow Horse Brave Heart M, Chase J, Elkins J, et al. Historical trauma among indigenous peoples of the Americas: Concepts, research, and clinical considerations. J Psychoactive Drugs. 2011;43(4):282–290; doi: 10.1080/02791072.2011.628913 22400458

[13] Wabanaki Public Health and Wellness. WPHW Definitions. Internal Correspondence Sent; January 2, 2022.

[14] Loftus S. Racist Sign on Millinocket Storefront Sparks Backlash. Bangor Daily News; June 21, 2022. Available from: https://www.bangordailynews.com/2022/06/21/news/penobscot/millinocket-storefront-racist-sign/ [Last accessed: August 2022].

[15] Russell L. A Healing Circle Will Let Millinocket Residents Air Distress About Business' Racist Sign. Bangor Daily News; June 30, 2022. Available from: https://www.bangordailynews.com/2022/06/30/news/penobscot/millinocket-racism-healing-circle-joam40zk0w/ [Last accessed: August 2022].

